# Potentially inappropriate prescribing in older hospitalized Dutch patients according to the STOPP/START criteria v2: a longitudinal study

**DOI:** 10.1007/s00228-020-03052-2

**Published:** 2020-12-02

**Authors:** Birgit A. Damoiseaux-Volman, Stephanie Medlock, Kimmy Raven, Danielle Sent, Johannes A. Romijn, Nathalie van der Velde, Ameen Abu-Hanna

**Affiliations:** 1 Amsterdam UMC, Department of Medical Informatics, Amsterdam Public Health Research Institute, University of Amsterdam, Meibergdreef 9, Amsterdam, The Netherlands; 2 Amsterdam UMC, Department of Medicine, Amsterdam Public Health Research Institute, University of Amsterdam, Meibergdreef 9, Amsterdam, The Netherlands; 3 Amsterdam UMC, Section of Geriatric Medicine, Amsterdam Public Health Research Institute, University of Amsterdam, Meibergdreef 9, Amsterdam, The Netherlands

**Keywords:** Inappropriate prescribing, Aged, Hospital, Older patients, Polypharmacy

## Abstract

**Purpose:**

To investigate prevalence, independent associations, and variation over time of potentially inappropriate prescriptions in a population of older hospitalized patients.

**Methods:**

A longitudinal study using a large dataset of hospital admissions of older patients (≥ 70 years) based on an electronic health records cohort including data from 2015 to 2019. Potentially inappropriate medication (PIM) and potential prescribing omission (PPO) prevalence during hospital stay were identified based on the Dutch STOPP/START criteria v2. Univariate and multivariate logistic regression were used for analyzing associations and trends over time.

**Results:**

The data included 16,687 admissions. Of all admissions, 56% had ≥ 1 PIM and 58% had ≥ 1 PPO. Gender, age, number of medications, number of diagnoses, Charlson score, and length of stay were independently associated with both PIMs and PPOs. Additionally, number of departments and number of prescribing specialties were independently associated with PIMs. Over the years, the PIM prevalence did not change (OR = 1.00, *p* = .95), whereas PPO prevalence increased (OR = 1.08, *p* < .001). However, when corrected for changes in patient characteristics such as number of diagnoses, the PIM (aOR = 0.91, *p* < .001) and PPO prevalence (aOR = 0.94, p < .001) decreased over the years.

**Conclusion:**

We found potentially inappropriate prescriptions in the majority of admissions of older patients. Prescribing relatively improved over time when considering complexity of the admissions. Nevertheless, the high prevalence shows a clear need to better address this issue in clinical practice. Studies seeking effective (re)prescribing interventions are warranted.

**Supplementary Information:**

The online version contains supplementary material available at 10.1007/s00228-020-03052-2.

## Introduction

Prescription of medications for older people is complex due to multimorbidity, polypharmacy, and age-specific changes in pharmacokinetics and pharmacodynamics [1, 2]. Most clinical guidelines contain treatment advice for single diseases and provide insufficient support for prescription of multiple drugs in older populations with multiple diseases [3, 4]. Potentially inappropriate prescribing includes misprescribing (e.g., drug choice: better alternatives), overprescribing (e.g., no clear indication), and underprescribing (e.g., no preventative medications) and can be categorized as potentially inappropriate medications (PIMs) or potential prescribing omissions (PPOs) [5–8]. Potentially inappropriate prescribing is common in older hospitalized patients with a PIM prevalence varying from 23 to 77% and PPO prevalence varying from 51 to 73% [9]. A longitudinal study of 44 general practices showed that the likelihood of potentially inappropriate prescribing is higher after hospitalization compared with before [10]. PIMs are associated with adverse patient-related outcomes, such as hospitalization and adverse drug events (ADEs) [11, 12]. In a meta-analysis pooling data from studies with a “new-user design” (implying that patients did not receive the treatment prior start of the study), PIMs were also associated with increased mortality (RR = 1.59) [13].

Several tools have been developed to identify potentially inappropriate prescriptions and help physicians and pharmacists improve prescribing in older patients. A systematic review found 42 tools of which 79% gave advice on medications that could potentially be stopped and 29% on medications that could potentially be started [12]. Another systematic review, searching in a broader time span, identified 73 different tools [14]. Both reviews report the effect of potentially inappropriate prescribing identified by STOPP/START criteria on clinically relevant outcomes. In the first review, PIMs or PPOs identified by the Beers and STOPP/START criteria were associated with six adverse patient-related outcomes, such as falls and functional decline [12]. In the second review, interventions with the STOPP/START criteria and Fit fOR The Aged (FORTA) had both a positive effect on clinical endpoints (compared to the control group) as shown in two RCTs each [14]. The STOPP/START criteria were updated and a second version was published [5]. This second version includes more criteria, advice on alternative therapies, and special considerations of use [15]. In a secondary analysis comparing the first and second versions of STOPP/START, more PIMs and PPOs were associated with preventable medication-related admissions (23% vs 40% of all admissions, *p* < 0.001) in version 2 [16].

A systematic review and recently published study show that studies conducted with the STOPP/START criteria v2 in hospitalized patients thus far have included relatively small sample sizes (between 72 and 319 patients, except one study with 1900 patients) [16, 17]. Furthermore, the sample sizes were much smaller for hospitalized patients (*n* = 3964 in 15 studies) compared to community care (*n* = 1,242,010 in 15 studies) [17]. Additional studies with larger sample sizes are needed to give better insight into the prevalence of potentially inappropriate prescribing in hospitalized patients according to the updated version of the STOPP/START criteria. Longitudinal studies are especially of interest to detect variations in prevalence over time. The variation of PIMs and PPOs over time is understudied in this population. Therefore, in the present study, we aimed to investigate potentially inappropriate prescribing in older patients during hospital stay according to the STOPP/START criteria v2, including prevalence, independent associations, and trends over time, using a large dataset based on an electronic health records (EHR) cohort.

## Methods

### Study population and data collection

The setting was a 1002-bed university medical center located in Amsterdam, the Netherlands. Data were derived from the hospital EHR and included admissions of older patients (≥ 70 years). We selected this age criterion based on Dutch guidelines for care of older inpatients (e.g., polypharmacy) [18]. Although our research question was retrospectively defined, the data was prospectively collected for each patient in the hospital EHR. The data contained diagnoses, gender, age, admission/discharge dates, medication orders and administrations, laboratory results, blood pressures, fall risk and delirium risk scores. Inclusion criteria were hospital admissions of ≥ 24 h to one of 26 clinical departments, with admission dates ≥ 1 November 2015 and discharge dates < 1 November 2019 (4 full years).

### Technical translation of the STOPP/START

PIMs and PPOs were identified based on the Dutch STOPP/START v2 [19]. The consensus study of the original STOPP/START v2 by Huibers et al. was used to define the technical translation [20]. Differences in technical translation between Huibers et al. and our script can be found in Appendix 1. In summary, we applied 102 of the 108 Dutch criteria, of which 457 required a modification of the translation provided by Huibers et al.

The scripts to code the PIMs and PPOs were constructed by the first author (BD) and checked by a medical computer scientist (AA). The scripts were manually checked using a random sample of patients by two researchers (BD and KR). R-studio (version 1.2.1335) with the packages dplyr, readr, lubridate, stringr, openxlsx, comorbidity, table 1, geepack, ggplot2, grid, and gridExtra was used for the data scripts, analysis, and visualization.

### Statistical analysis

Prevalence of potentially inappropriate prescribing was expressed as the percentage of hospital admissions with at least one PIM or PPO during hospital stay. Additionally, individual PIMs and PPOs were calculated as a percentage of all admissions. We used univariate and multivariate logistic regression to investigate variables associated with PIMs and PPOs and to observe the trends over time in the prevalence of PIMs and PPOs. All analyses were adjusted for clustered data (patients admitted multiple times) using generalized estimating equations (GEE).

We selected eight variables to investigate associations with PIMs and PPOs: age, gender, number of medications, number of diagnoses, Charlson score, length of stay, number of departments (where a patient was admitted during one admission), and number of different prescribing specialties (during one admission). In the multivariate models, the eight variables were corrected for confounders or covariates. We constructed directed acyclic graphs per variable to define these confounders and covariates. The average predictive comparison (APC) was calculated for the variables by obtaining the difference between the mean predicted outcome with the variable value set at its 75th percentile and the mean predicted outcome with the variable value set at its 25th percentile [21]. For the binary variable gender, the APC is the mean predicted outcome when gender is set to “female” for all patients, minus the mean predicted outcome when gender is set to “male” for all patients.

We calculated the prevalence over time of admissions with ≥ 1 PIM or ≥ 1 PPO and of admissions with the top 5 most common (based on overall prevalence) PIMs or PPOs. We corrected for patient characteristics by adjusting for eight covariates, aside from time, in the multivariate models. We used a *p* value of < 0.01 as significant in all analyses. For visualization of PIMs and PPOs over time, we predicted the probability of having a PIM or PPO per admission (not per patient) and plotted this prediction over time.

### Ethics approval

The Medical Ethics Review Committee of our university medical center reviewed the study plan and determined that according to the Medical Research Involving Human Subjects Act (WMO), official approval was not required (reference number W18_027#18.043).

## Results

### Characteristics

The overall number of hospital admissions (≥ 24 h) of older patients (≥ 70 year) was 16,687 during 4 years (Table 1). The patients had a mean age of 77.2 at time of hospital admission.

**Table 1:** Characteristics of admissions

	Admissions *N* = 16,687*
Gender, % (*n*)	
Male	52.4 (8744)
Female	47.6 (7943)
Age, mean (SD)	77.2 (5.76)
Length of stay in days, median (IQR)	4.1 (2.0–8.1)
Died during admission, % (*n*)	5.0 (840)
Number of medications during hospital stay^†^, median (IQR)	16.0 (10.0–24.0)
Top 10 administrated drugs (ATC level 2), % (*n*)	
B01–antithrombotic agents	85.4 (14,257)
B05–blood substitutes and perfusion solutions	77.9 (13,000)
N02–analgesics	76.2 (12,722)
A02–drugs for acid related disorders	69.0 (11,506)
J01–antibacterials for systemic use	64.3 (10,738)
N01–anesthetics	56.0 (9337)
C01–cardiac therapy	54.4 (9070)
C10–lipid modifying agents	44.8 (7471)
C07–beta blocking agents	44.6 (7439)
N05–psycholeptics	44.1 (7358)
Number of diagnoses, median (IQR)	5.0 (3.0–7.0)
Weighted Charlson score, median (IQR)	1.0 (0.0–3.0)
Comorbidities or risk factors, % (*n*)	
Risk of falling	52.7 (8794)
Hypertension	36.9 (6163)
Malignant neoplasm	32.7 (5456)
Diabetes mellitus	22.7 (3783)
Delirium risk (DOSS ≥ 3)	13.2 (2206)
Asthma/COPD	11.4 (1903)
Heart failure	9.2 (1538)
Low kidney function (< 50 ml/min) ^ǂ^	7.5 (1251)
Dementia	2.4 (397)
Atrial fibrillation	1.3 (211)
Depression	0.3 (53)
Number of departments per hospital stay, median (IQR)	2.0 (1.0–3.0)
Number of prescribing specialties, median (IQR)	3.0 (2.0–4.0)

*SD*, standard deviation; *IQR*, interquartile range

*11,289 unique patients

^†^Number of unique ATC codes (level 5–chemical substance) per admission

^ǂ^Kidney function was reported as eGFR calculated by either MDRD or CKD-EPI

### Prevalence of PIMs and PPOs

Table 2 shows that of all admissions, 55.5% had ≥ 1 PIM and 58.1% had ≥ 1 PPO. The most common PIM was “Benzodiazepines with history or risk of falls” (22.1%), and the most common PPO was “laxatives in patients receiving opioids” (17.3%). Prevalence of all individual PIMs and PPOs can be found in Appendix 1. All PIMs in the categories “renal,” “gastrointestinal,” and “urogenital” and all PPOs in the categories “central nervous & ophthalmic” and “influenza vaccine” had a prevalence below 1%.

**Table 2:** Prevalence PIMs and PPOs as percentage of all admissions

	Admissions *N* = 16,687
At least 1 PIM, % (n)	55.5 (9268)
1 PIM	27.1 (4526)
2 PIMs	15.1 (2522)
3 PIMs	7.3 (1211)
4 or more PIMs	6.0 (1009)
At least 1 PPO, % (*n*)	58.1 (9695)
1 PPO	29.9 (4986)
2 PPOs	15.5 (2580)
3 PPOs	7.4 (1230)
4 or more PPOs	5.4 (899)
Top 5 PIM, % (*n*)	
1 Benzodiazepines with history or risk of falls	22.1 (3683)
2 Loop diuretic as treatment for hypertension	11.0 (1838)
3 Neuroleptic drugs with history or risk of falls	10.2 (1704)
4 Antiplatelet agents with vitamin K antagonist, direct thrombin inhibitor or factor Xa inhibitors in patients with stable coronary, cerebrovascular or peripheral arterial disease	8.0 (1335)
5 Centrally-acting antihypertensives	6.1 (1011)
Top 5 PPO, % (*n*)	
1 Laxatives in patients receiving opioids	17.3 (2881)
2 PPI with a low dose acetylsalicylic acid or carbasalate calcium (and age-specific criteria)	16.7 (2780)
3 ACE inhibitor (or angiotensin receptor blocker in case of side effects ACE inhibitor) with systolic heart failure and/or documented coronary artery disease	16.1 (2686)
4 Statin therapy with a documented history of coronary, cerebral or peripheral vascular disease and LDL > 2,5 mmol/l, unless the patient’s status is end-of-life (life expectancy < 3 years)	10.2 (1693)
5 Appropriate beta-blocker with stable systolic heart failure	9.2 (1538)

*ACE*, angiotensin-converting enzyme; *PPI*, proton pump inhibitor

### Associations with PIMs and PPOs

We used 16 univariate logistic models (per variable, one model for PIMs and one model for PPOs) and 16 multivariate logistic models to analyze the associations with PIMs and PPOs. Table 3 shows that all variables were independently associated with PIMs in the multivariate models. Gender, age, number of medications, number of diagnoses, Charlson score, and length of stay were independently associated with PPOs. Analysis of the APC showed that number of medications (APC = 0.34) was a strong predictor of PIMs and number of diagnoses (APC = 0.17) of PPOs.

**Table 3:** Associations with PIMs and PPOs

	PIMs			PPOs		
	Crude OR (95% CI)	Adjusted^ǂ^ OR (95% CI)	Adjusted APC	Crude OR (95% CI)	Adjusted^ǂ^ OR (95% CI)	Adjusted APC
Gender (Female vs male)	1.12 (1.04-1.20)*	1.11 (1.03-1.19)*	0.03	0.68 (0.63–0.73)†	0.67 (0.62–0.72)†	0.10
Age^§^	1.07 (1.06–1.08)^†^	1.07 (1.06–1.07)^†^	0.03	1.05 (1.04–1.06)^†^	1.07 (1.06–1.07)^†^	0.03
Number of medications	1.11 (1.11–1.11)^†^	1.12 (1.11–1.13)^†^	0.34	1.02 (1.02 – 1.02)^†^	1.02 (1.02–1.03)^†^	0.06
Number of diagnoses	1.24 (1.22–1.25)^†^	1.24 (1.22–1.25)^†^	0.21	1.19 (1.18 –1.21)^†^	1.19 (1.17–1.20)^†^	0.17
(Weighted) Charlson score	1.09 (1.08–1.11)^†^	1.10 (1.08 – 1.11)^†^	0.07	1.07 (1.06–1.09)^†^	1.07 (1.06–1.09)^†^	0.05
Length of stay^§^	2.00 (1.98–2.02)^†^	1.53 (1.51–1.54)^†^	0.18	1.03 (1.02–1.03)*	0.86 (0.85–0.86)^†^	0.10
Number of departments	1.50 (1.45–1.54)^†^	1.60 (1.55–1.66)^†^	0.20	1.04 (1.01–1.07)*	1.03 (1.00–1.07)	0.01
Number of prescribing specialties	1.49 (1.46–1.53)^†^	1.10 (1.07–1.14)^†^	0.04	1.10 (1.08–1.13)^†^	1.02 (0.99–1.05)	0.01

APC, average predictive comparison

*p value < 0.01

^†^*p* value < 0.001

^ǂ^Gender was adjusted for age | Age was adjusted for gender | Number of medications was adjusted for age, length of stay, number of diagnoses, Charlson score, gender, number of departments, number of prescribing specialties | Number of diagnoses was adjusted for age and gender | Charlson score was adjusted for age and gender | Length of stay was adjusted for age, number of diagnoses, Charlson score, gender, number of departments | Number of department was adjusted for age, number of diagnoses, Charlson score, gender | Number of prescribing specialties was adjusted for age, length of stay, number of diagnoses, Charlson score, gender, number of departments

^§^Age: per 5 years | Length of stay: per 5 days

### Time trends

**Fig. 1:**
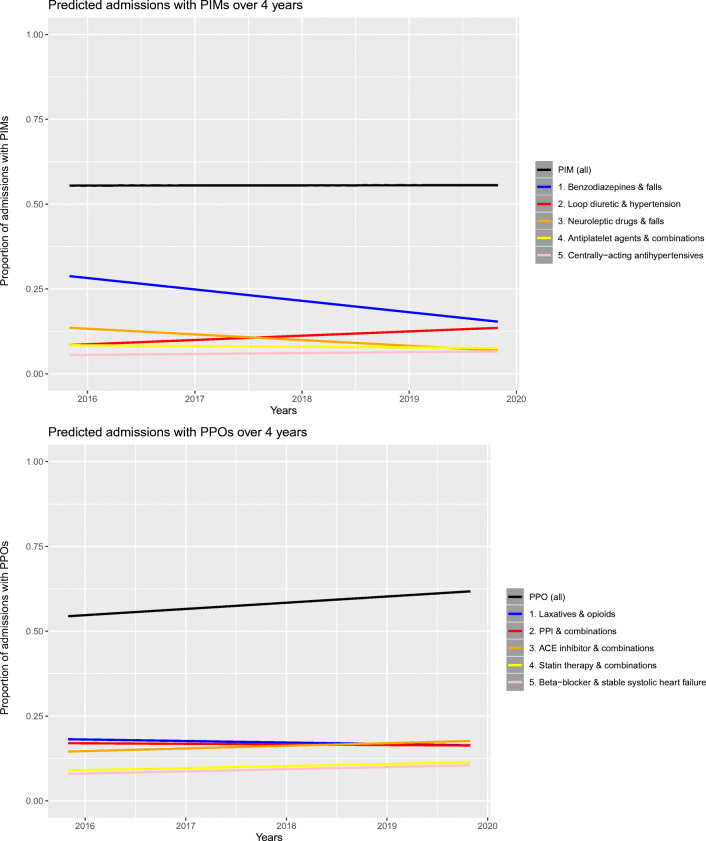
Time trends in the percentage of admissions with PIMs or PPOs (unadjusted models)

Figure 1 shows the time trends for admissions with ≥ 1 PIM, ≥ 1 PPO, and a top 5 most prevalent PIM/PPO. All ORs and aORs for prevalence of PIMs, PPOs, and the top 5 PIMs or PPOs over time can be found in Appendix 2.

For PIMs overall, no significant change per year was found in the unadjusted models (OR = 1.00, CI = 1.00–1.00, *p* = 0.95). Of the top 5 PIMs, two PIMs (benzodiazepines & falls; neuroleptic drugs & falls) declined, one PIM (loop diuretic for hypertension) increased, and two PIMs (antiplatelet agents & combinations; centrally acting antihypertensives) had no change in prevalence over the years. A significant increase of admissions with ≥ 1 PPO per year was found in the unadjusted analysis (OR = 1.08, CI = 1.08–1.08, *p* < 0.001). Of the top 5 PPOs, three PPOs increased (ACE inhibitor & combinations; statin therapy & combinations; beta-blocker & stable systolic heart failure) and two PPOs (laxatives & opioids; proton pump inhibitor & combinations) did not change over the years.

Admissions with ≥ 1 PIM and admissions with ≥1 PPO significantly decreased over the years in the adjusted models (PIM aOR = 0.91, CI = 0.91–0.91, *p* < 0.001; PPO aOR = 0.94, CI = 0.94–0.94, *p* < 0.001).

## Discussion

We found that more than half of all hospital admissions of older patients (> 70 years) had a PIM (55.5%) or PPO (58.1%). Gender, age, number of medications, number of diagnoses, Charlson score, and length of stay were independently associated with both PIMs and PPOs. Additionally, number of departments and number of prescribing specialties were independently associated with PIMs. Although the absolute PIM prevalence remained the same and the absolute PPO prevalence increased over the years, the PIM and PPO prevalence decreased over the years when corrected for patient characteristics, such as number of medications and number of diagnoses.

A major strength of our study is that we had a very large dataset with more than 16,000 hospital admissions for the analyses. This allowed us to investigate the association of eight variables and prevalence over time. In addition, we could apply almost all of the Dutch STOPP/START criteria v2 (102/108). For our analysis, we used medication administration data, which informs about medications actually consumed with very precise timing. Nurses record the medication given by scanning the barcode of the patient and medication. The diagnosis data that we used is manually extracted and coded for a national registry for quality purposes, and gives a highly accurate list of the diagnoses relevant to the admission (main diagnoses, co-morbidities and complications). Limitations of our study include that diagnosis data may have incomplete documentation of co-morbidity not relevant to the admission and therefore the prevalence of PIMs and PPOs may have been underestimated. Because we used routinely collected data, we could not establish whether clinicians did not follow STOPP/START criteria for clinical reasons such as side effects. We studied admissions of one hospital, and therefore, the results are not directly generalizable to other hospitals and other countries.

The PIM (56%) and PPO (58%) prevalence in our study was comparable to that found in other studies in hospital settings. A recent systematic review found a PIM rate of 52% and PPO rate of 64% for in total 3964 hospitalized patients over 15 studies which, like our study, used the STOPP/START v2 [17]. A study of hospitalized patients in Spain found a PIM prevalence of 61% using STOPP/START v1, and 51% using Beers, and a PPO prevalence of 51% using STOPP/START v1 and 57% using ACOVE 3. The differences with our results could be due to the populations, study size, or differences in the instruments used [22]. To our knowledge, our study is the first to assess for trends in PIM and PPO prevalence over time for in-hospital patients. Longitudinal studies have been conducted for non-hospitalized patients; in an Irish community-dwelling cohort, the prevalence of PPOs and PIMs increased over time (not significant in multivariable model) and in a Dutch study assessing patients of general practitioners, PIM prevalence did not change and PPO decreased over time [23, 24]. These differences with our findings may be explained by different settings (hospital vs community-dwelling) and/or by differences in covariates (age and gender in the community-dwelling Dutch study; the Irish study added number of regular medications and chronic conditions). However, the differences in results between the Dutch and Irish population-based cohort studies suggest that there could be between-country differences in longitudinal PIM and PPO prevalence as well [23, 24].

Our results showed that number of medications, number of diagnoses, Charlson score, and age were independently associated with a higher prevalence of PIMs and PPOs. Number of medications was also an important predictor for PIMs according to the analysis of APC. In multiple studies, the number of medications was also associated with PIMs in hospitalized patients [22, 25, 26]. A recent qualitative study found that a large number of medications was the most frequently mentioned barrier in prescribing for older patients [27]. The median number of medications in our study was 16.0, which is relatively high compared to other studies with hospitalized patients [17]. This difference could be attributable to the complexity of patients typically admitted to our university medical center, a tertiary referral center, and/or due to our decision to include all administrated medications with an ATC code at level 5 (including short-term medications). Number of diagnoses was an important predictor for PPOs according to the analysis of APC in our study. Comorbidity burden has been shown to be significantly associated with PIMs in frail patients [25]. The relationship between number of diagnoses and PIMs and PPOs has not been frequently studied, and therefore, this finding is of interest for future studies.

In contrast to our findings, age was not associated with PIMs in a previous study using STOPP/START v2 [26]. This difference can be explained by the number of patients (319 patients vs 16,687 admissions), age of included patients (> 50% were below 70 years), and differences in covariates.

Interestingly, our results showed that female gender (compared to male gender) and length of stay were independently associated with a higher prevalence of PIMs and lower prevalence of PPOs. In previous studies, gender was inconsistently associated with PIMs [25, 26, 28]. However, in line with our findings, two studies indicated that females had a higher risk of having a PIM [29, 30]. In contrast with our findings, in previous studies, length of stay was not a significant risk factor for PIMs [26, 31]. However, this can be explained by differences in number of included patients, age (> 60 years), departments, and covariates. We found that multiple prescribing specialties and multiple departments were independently associated with PIMs, but not with PPOs. In another study, the variable “multiple prescribers” was associated with inappropriate drug use at hospital discharge [32].

We chose to use only data from admissions ≥ 24 h. Our hospital admissions of < 24 h include day-clinic appointments (e.g., chemotherapy) which are a type of outpatient clinic appointments and, in our opinion, form a different population than hospital inpatients. Furthermore, we excluded emergency admissions for which the diagnosis data would be of lower quality. Inappropriate prescribing in the outpatient clinic and short-term (emergency) admissions is also important, and is a good topic for further research. Polypharmacy and inappropriate prescriptions in geriatric oncology patients might be influenced by the management of cancer [33]. Sub-analysis in our data of the admissions with a cancer diagnosis (32.7%) shows a lower prevalence of PIMs (52.5%) and PPOs (48.8%), compared to the total population. Reasons for a lower prevalence and associations with clinically relevant outcomes should be investigated in follow-up research for this sub-population.

To improve prescribing for older patients, five clinical trials have evaluated interventions with the STOPP/START criteria v1 and showed improvements in adverse drug reactions, falling, medication costs, and appropriateness [34]. In line with these studies, we are currently developing an intervention including a clinical decision support system (CDSS) based on the STOPP/START criteria v2 for older hospitalized patients with a high risk of falls or delirium to be used at point of care.

## Conclusions and implications

In conclusion, we assessed the prevalence of PIMs (55.5%) and PPOs (58.1%) in hospitalized older patients in an academic hospital. Over time, the absolute percentage of admissions with ≥ 1 PIM was stable and ≥ 1 PPO increased. However, the admissions with PIMs and PPOs decreased over the years when corrected for changes in patient characteristics, such as number of diagnoses. Therefore, we can conclude that prescribing relatively improved for older hospitalized patients when considering the complexity of the admissions. Nevertheless, the high prevalence of PIMs and PPOs in older in-hospital patients shows a clear need to better address this issue in clinical practice. Studies seeking effective (re)prescribing interventions are warranted.

## Supplementary Information

ESM 1 (DOCX 58.4 kb)

## Data Availability

The datasets used during the current study were collected for healthcare purposes and not for research. The data is potentially available via the corresponding author after consent of the patients and/or in anonymized form. However, data analysis for current study is not possible with anonymized data. Requests for data will be subject to review by the Amsterdam UMC research data management.
