# Supplementary material for: Potentially inappropriate prescribing in older hospitalized Dutch patients according to the STOPP/START criteria v2: a longitudinal study
Source: Eur J Clin Pharmacol. 2020 Dec 2;77(5):777–85. doi: 10.1007/s00228-020-03052-2 (PMC8032616; doi:10.1007/s00228-020-03052-2)
Supplement: Supplementary file 1 — (DOCX 58.4 kb) [file 228_2020_3052_MOESM1_ESM.docx]

**Appendices**

Potentially inappropriate prescribing in older hospitalized Dutch patients according to the STOPP/START criteria v2: a longitudinal study

**Appendix 1: Technical translation and prevalence Dutch STOPP/START v2**

| Table A1. Technical translation and prevalence (as a percentage of all admissions) per Dutch STOPP criteria v2 | | | | |
| --- | --- | --- | --- | --- |
|  | STOPP criteria | Technical translation* | % ** | % time*** |
|  | Any drug prescribed without an evidence-based clinical indication. | Not coded (as Huibers et al) | - | - |
|  | Any drug prescribed beyond the recommended duration, where treatment duration is well defined. | Not coded (as Huibers et al) | - | - |
|  | Any duplicate drug class prescription | Not coded (as Huibers et al) | - | - |
| B. Cardiovascular system | Digoxin for heart failure with preserved systolic ventricular function | Huibers et al  *Data “left ventricular ejection fraction” not available* | 0.58 | - |
|  | Verapamil or diltiazem with NYHA Class III or IV heart failure | Huibers et al | 0.37 | - |
|  | Beta-blocker in combination with verapamil or diltiazem | Huibers et al | 1.09 | - |
|  | Beta blocker with symptomatic bradycardia, type II heart block or complete heart block | Huibers et al  *Data heart rate not available* | 1.47 | - |
|  | Amiodarone as first-line antiarrhythmic therapy | Huibers et al | 2.48 | - |
|  | Loop diuretic as treatment for hypertension | Huibers et al | 11.01 | - |
|  | Loop diuretic for dependent ankle oedema without clinical, biochemical evidence or radiological evidence of heart failure, liver failure, nephrotic syndrome or renal failure | Huibers et al | 0.21 | - |
|  | Thiazide diuretic with current significant hypokalaemia, hyponatraemia, hypercalcaemia or with a history of gout | Huibers et al  *We used labdata* | 0.80 | - |
|  | Centrally-acting antihypertensives | Huibers et al | 6.06 | - |
|  | ACE inhibitors or angiotensin Receptor Blockers in patients with hyperkalaemia | Huibers et al  *We used labdata* | 0.95 | - |
|  | Aldosterone antagonists with concurrent potassium-conserving drugs without monitoring of serum potassium | Huibers et al  *Adjusted time monitoring of potassium due to available data* | 5.45 | - |
|  | Phosphodiesterase type-5 inhibitors in severe heart failure characterized by hypotension or concurrent daily nitrate therapy for angina | Huibers et al | 0.08 | - |
| C. Coagulation system | Antiplatelet agents at doses greater than 80 or 100 mg per day (with exception of the first dose) | Huibers et al  *Dutch v2 has different doses and durations* | 0.90 | - |
|  | Antiplatelet agents, clopidogrel and other drugs from the same group, dipyridamole, vitamin K antagonists, direct thrombin inhibitors or factor Xa inhibitors with concurrent significant bleeding risk or recent relevant spontaneous bleeding | Huibers et al | 1.50 | - |
|  | Antiplatelet agents plus clopidogrel (or other drugs from the same group) as secondary stroke prevention, unless the patient has a coronary stent(s) inserted in the previous 12 months or concurrent acute coronary syndrome or has a high grade symptomatic carotid arterial stenosis | Huibers et al | 1.46 | - |
|  | Antiplatelet agents in combination with vitamin K antagonist, direct thrombin inhibitor or factor Xa inhibitors in patients with chronic atrial fibrillation | Huibers et al | 0.20 | - |
|  | Antiplatelet agents with vitamin K antagonist, direct thrombin inhibitor or factor Xa inhibitors in patients with stable coronary, cerebrovascular or peripheral arterial disease | Huibers et al  *Removed ICD9 36.0, this gave other results (glaucoma)* | 8.00 | - |
|  | Vitamin K antagonist, direct thrombin inhibitor or factor Xa inhibitors for first deep venous thrombosis without continuing provoking risk factors for > 6 months | Huibers et al | 0.00 | 0.00 |
|  | Vitamin K antagonist, direct thrombin inhibitor or factor Xa inhibitors for first pulmonary embolus without continuing provoking risk factors for > 12 months | Huibers et al | 0.00 | 0.00 |
|  | NSAID and vitamin K antagonist, direct thrombin inhibitor or factor Xa inhibitors in combination | Huibers et al | 0.78 | - |
| D. Central Nervous system | Tricyclic antidepressants with dementia, untreated narrow angle glaucoma, cardiac conduction abnormalities, prostatism, Sjogren's illness or previous urinary retention | Huibers et al | 0.21 | - |
|  | Tricyclic antidepressants as first-line antidepressant treatment | Huibers et al | 0.09 | - |
|  | Neuroleptics with moderate-marked anticholinergic effects with prostatism or previous urinary retention | Huibers et al | 0.01 | - |
|  | SSRI’s and non-iatrogene hyponatraemia i.e. serum Na+ < 130 mmol/l in last 2 months | Huibers et al | 0.16 | - |
|  | Benzodiazepines for ≥ 4 weeks | Huibers et al | 1.25 | 35.30 |
|  | Antipsychotics (i.e. other than quetiapine or clozapine) in those with parkinsonism | Huibers et al | 0.08 | - |
|  | Anticholinergics to treat extra-pyramidal side-effects of neuroleptic medications | Huibers et al | 0.03 | - |
|  | Medications with anticholinergic effects in patients with delirium or dementia | Huibers et al | 1.78 | - |
|  | Antipsychotics in patients with behavioural and psychological symptoms of dementia unless symptoms are severe and not medical treatments have failed | Huibers et al | 0.83 | - |
|  | Neuroleptics as hypnotics | Huibers et al | 0.01 | - |
|  | Acetylcholinesterase inhibitors with bradycardia (< 60 beats/min.), heart block or recurrent unexplained syncope | Huibers et al | 0.07 | - |
|  | Phenothiazines with exception of chloorpromazin for hiccoughs and levopromazine in palliative care | Huibers et al | 0.02 | - |
|  | Levodopa or dopamine agonists for benign essential tremor | Huibers et al | 0.03 | - |
|  | First-generation antihistamines | Huibers et al | 2.80 | - |
| E. Renal system | Digoxin at a dose > 0.125mg/day if eGFR < 30 ml/min/1.73m2 | Huibers et al  W*e used labdata* | 0.00 | - |
|  | Direct thrombin inhibitors if eGFR < 30 ml/min/1.73m2 | Huibers et al - *We used labdata* | 0.07 | - |
|  | Factor Xa inhibitors if eGFR < 15 ml/min/1.73m2 | Huibers et al - *We used labdata* | 0.11 | - |
|  | NSAID’s if eGFR < 30 ml/min/1.73m2 | Huibers et al  *We used labdata & in Dutch v2 different GFR* | 0.08 | - |
|  | Metformin if eGFR < 30 ml/min/1.73m2 | Huibers et al - *We used labdata* | 0.82 | - |
|  | Bisphosphonates if eGFR < 30 or 50 ml/min/1.73m2 | Technical translation by our study group - *Criterion not in Huibers et al* | 0.00 | - |
| F. Gastrointestinal | Metoclopramide with Parkinsonism | Huibers et al  *Dutch v2 differs in medications* | 0.06 | - |
|  | PPI for peptic ulcer disease or oesophagitis with exception of barrett's oesophagus at full therapeutic dosage for > 8 weeks | Huibers et al  *Dutch v2 differs in diagnoses* | 0.34 | 44.44 |
|  | Drugs likely to cause or worsen constipation in patients with chronic constipation | Huibers et al | 0.52 | - |
|  | Iron preparations with  regulated release or oral elemental iron doses greater than 200 mg daily | Huibers et al | 0.08 | - |
| G. Respiratory | Theophylline as monotherapy for COPD | Huibers et al | 0.02 | - |
|  | Systemic corticosteroids instead of inhaled corticosteroids for maintenance therapy in moderate-severe COPD or asthma | Huibers et al  *Dutch v2 differs in diagnoses* | 4.57 | - |
|  | Anti-muscarinic bronchodilators (e.g. ipratropium, tiotropium) with untreated narrow angle glaucoma or bladder outflow obstruction | Huibers et al | 0.32 | - |
|  | Stop benzodiazepines with acute or chronic respiratory failure i.e. pO2 < 8.0 kPa ± pCO2 > 6.5 | Not coded  *Data for diagnoses not available* | - | - |
| H. Musculoskeletal system | NSAID with moderate-severe hypertension or heart failure | Huibers et al | 2.18 | - |
|  | Long-term use of NSAID (>3 months) for symptom relief of osteoarthritis pain where paracetamol has not been tried | Huibers et al  *Dutch v2 contains extra information about dosing – consensus needed - therefore extra information not coded* | 0.00 | 0.00 |
|  | Long-term corticosteroids (>3 months) as monotherapy for rheumatoid arthritis | Huibers et al | 0.00 | 0.00 |
|  | Corticosteroids (other than periodic intra-articular injections for mono-articular pain) for osteoarthritis | Huibers et al | 0.77 | - |
|  | Long-term NSAID or colchicine for chronic treatment of gout where there is no contraindication to a xanthine-oxidase inhibitor | Huibers et al | 0.00 | 0,00 |
|  | COX-2 selective NSAIDs and diclofenac with concurrent cardiovascular disease | Huibers et al  *Dutch v2 differs in medications* | 0.47 | - |
|  | Oral bisphosphonates in patients with a history of upper gastrointestinal disease or in patients who stay in bed | Huibers et al  *Dutch v2 differs in diagnoses (with “patients who stay in bed”). Not coded due to in-hospital population.* | 0.10 | - |
| I. Urogenital | Anticholinergics for neurogenic bladder with concurrent dementia, chronic cognitive impairment, narrow-angle glaucoma or chronic prostatism | Huibers et al | 0.56 | - |
|  | Selective alpha-1 blockers in those with daily incontinence, symptomatic orthostatic hypotension, micturition syncope or urinary catheter in situ > 2months | Huibers et al  *Dutch v2 differs in diagnoses & data for urinary catheter in situ not available* | 0.07 | - |
| J. Endocrine system | Sulphonylureas with a long duration of action and active metabolites with type 2 diabetes mellitus | Huibers et al | 1.41 | - |
|  | Thiazolidenediones in patients with documented heart failure | Huibers et al | 2.35 | - |
|  | Beta-blockers in diabetes mellitus with frequent hypoglycaemic episodes | Huibers et al  *Data for “frequent” not available* | 0.17 | - |
|  | Oestrogens with a history of breast cancer or venous thromboembolism | Huibers et al | 0.01 | - |
|  | Oral oestrogens without progestogen in patients with intact uterus | Huibers et al | 0.35 | - |
|  | Androgens in the absence of primary or secondary hypogonadism | Huibers et al | 0.06 | - |
| K. Fall risk | Benzodiazepines with history or risk of falling | Huibers et al  *We used the fall risk score* | 22.07 | - |
|  | Neuroleptic drugs with history or risk of falling | Huibers et al  *We used the fall risk score* | 10.21 | - |
|  | Vasodilator drugs with orthostatic hypotension | Huibers et al | 0.12 | - |
|  | Hypnotic Z-drugs with history or risk of falling | Huibers et al | 2.81 | - |
| L. | Use of oral or transdermal strong opioids as first line therapy for mild pain | Huibers et al  *Data “NSAID in history” not available* | 4.87 | - |
| M/N. | Concomitant use of two or more drugs with antimuscarinic/anticholinergic properties | Huibers et al | 1.67 | - |

SSRI’s = Selective Serotonin Re-uptake Inhibitors, ACE= Angiotensin-Converting Enzyme, NSAID= Non-Steroidal Anti-Inflammatory Drugs

* ATC codes, ICD-9 and ICD-10 codes required for Dutch STOPP/START v2 were selected using Huibers et al or selected - by two researchers (BD and KR) - using [www.whocc.no/atc_ddd_index/](http://www.whocc.no/atc_ddd_index/), the Dutch medication information website [www.farmacotherapeutischkompas.nl/](http://www.farmacotherapeutischkompas.nl/), [www.icd9data.com](http://www.icd9data.com), [www.icd10data.com](http://www.icd10data.com) or diagnoses description in our data.

** Dutch STOPP/START criteria v2 with diagnoses were calculated over the whole admission. Criteria with only medications were calculated based on the medications administration dates and we identified a STOPP violation if two medications were administrated on the same day. We excluded medication administrations with missing values or status “planned” (indicating that the medication had not been given).

*** Prevalence (% time) was calculated as percentage of a selected population (i.e. female/male or admissions with a selected length of stay: ≥4 weeks in “Benzodiazepines for ≥ 4 weeks”).

| Table A2. Technical translation and prevalence (as a percentage of all admissions) per Dutch START criteria v2 | | | | |
| --- | --- | --- | --- | --- |
|  | START description | Technical translation* | % ** | % time*** |
| 1. Cardiovascular system | Vitamin K antagonists or direct thrombin inhibitors or factor Xa inhibitors in the presence of chronic atrial fibrillation (with exception of men 65-75 years without cardiovascular comorbidity) | Huibers et al  *Dutch v2 contains extra information - consensus needed - therefore extra information not coded* | 0.28 | - |
|  | Antiplatelet agents in the presence of chronic atrial fibrillation, where Vitamin K antagonists or direct thrombin inhibitors or factor Xa inhibitors are contraindicated or not wanted | Huibers et al | 0.90 | - |
|  | Acetyl salicylic acid or carbasalate calcium, clopidogrel, prasugrel or ticagrelor with a documented history of coronary, cerebral or peripheral vascular disease and sinus rhythm in patient not treated with Vitamin K antagonists or direct thrombin inhibitors or factor Xa inhibitors | Huibers et al  *Dutch v2 differs in medications* | 3.99 | - |
|  | Antihypertensive therapy where systolic blood pressure consistently > 160 mmHg and/or diastolic blood pressure consistently > 90 mmHg and lifestyle interventions have not enough effect; if systolic blood pressure > 140 mmHg and /or diastolic blood pressure > 90 mmHg, if diabetic | Huibers et al | 2.33 | - |
|  | Statin therapy with a documented history of coronary, cerebral or peripheral vascular disease or high cardiovascular risk and LDL > 2,5 mmol/l, unless the patient’s life expectancy < 3 years | Huibers et al  *No age limits & Dutch v2 contains extra information about diagnoses –consensus needed - therefore extra information not coded.* | 10.15 | - |
|  | ACE inhibitor (or angiotensin receptor blocker in case of side effects ACE inhibitor) with systolic heart failure and/or coronary artery disease | Huibers et al  *Dutch v2 differs in medications* | 16.10 | - |
|  | Beta-blocker with ischaemic heart disease or stable angina pectoris | Huibers et al | 7.75 | - |
|  | Appropriate beta-blocker with stable systolic heart failure | Huibers et al | 9.22 | - |
| 1. Respiratory | Inhaled beta 2 agonist or antimuscarinic bronchodilator for mild to moderate asthma or COPD | Huibers et al | 4.48 | - |
|  | Inhaled corticosteroid for COPD, where repeated exacerbations despite long-working bronchodilator | Huibers et al  *Dutch v2 differs in medications and diagnoses* | 0.18 | - |
|  | Continuous oxygen with documented chronic hypoxaemia | Not coded  *Data for diagnoses and oxygen not available* | - | - |
| 1. Central nervous & ophthalmic | Anti-Parkinson drug in idiopathic Parkinson’s disease with functional impairment and resultant disability | Huibers et al | 0.28 | - |
|  | Antidepressant drug in the presence of moderate-severe depressive symptoms | Huibers et al  *Dutch v2 differs in medications* | 0.06 | - |
|  | Acetylcholinesterase inhibitor for mild or moderate Alzheimer’s dementia or Lewy Body dementia | Huibers et al | 0.90 | - |
|  | Prostaglandin analogue or beta-blocker for primary open-angle glaucoma | Huibers et al  *Dutch v2 differs in medications* | 0.01 | - |
|  | SSRI’s (or SNRI or pregabalin if SSRI contraindicated) for persistent severe anxiety that interferes with independent functioning | Huibers et al | 0.14 | - |
|  | Dopamine agonist for severe restless legs syndrome with unacceptable suffering despite non-medical treatment, once iron deficiency and severe renal failure have been excluded | Huibers et al  *Dutch v2 contains extra information – consensus needed - therefore extra information not coded* | 0.02 | - |
| D. Gastrointestinal | Proton Pump Inhibitor with severe gastro-oesophageal reflux disease or peptic stricture requiring dilatation | Huibers et al | 0.08 | - |
|  | PPI with NSAID (and > 70 years) | Technical translation by our study group - *Criterion not in Huibers et al* | 0.58 | - |
|  | PPI with a low dose acetyl salicylic acid or carbasalate calcium (and age specific criteria) | Technical translation by our study group - *Criterion not in Huibers et al* | 16.66 | - |
|  | Fibre supplement for chronic diverticulosis with constipation | Huibers et al | 0.02 | - |
| E. Musculoskeletal system | DMARD with active, disabling rheumatoid disease (> 4 weeks) | Huibers et al  *Dutch v2 differs in diagnoses duration, data for “> 4 weeks” not available.* | 0.83 | - |
|  | Bisphosphonates and vitamin D and calcium in patients taking long-term systemic corticosteroid therapy (> 3 months) if dose ≥ 7.5 mg daily prednisone (or equivalent) | Huibers et al  *Dutch v2 differs in dosing.* | 0.02 | 12.50 |
|  | Vitamin D and calcium supplement in patients with osteoporosis | Huibers et al  *Dutch v2 differs on diagnoses.* | 0.92 | - |
|  | Bone anti-resorptive or anabolic therapy in patients with documented osteoporosis, where no contraindication exists | Huibers et al  *Dutch v2 differs on diagnoses.*  *No data on “T-score” available.* | 0.79 | - |
|  | Vitamin D supplement in older people who are housebound or experiencing falls or with osteopenia | Huibers et al  *No data on “T-score” available.* | 2.92 | - |
|  | Xanthine-oxidase inhibitors with a history of recurrent episodes of gout or gout tophi | Huibers et al  *No data on “recurrent” and “gout tophi available.* | 0.89 | - |
|  | Folic acid supplement in patients taking methotrexate | Huibers et al | 0.07 | - |
| F. endocrine | Metformin in type 2 diabetes mellitus (not if eGFR < 30 ml/min/1.73m2) | Technical translation by our study group - *Criterion not in Huibers et al* | 4.57 | - |
|  | ACE inhibitor or Angiotensin Receptor Blocker (if intolerant of ACE inhibitor) in diabetes with evidence of renal disease | Huibers et al  *No labdata on “microalbuminuria” available* | 0.74 | - |
| G. Urogenital | Alpha-1 receptor blocker with symptomatic prostatism, where prostatectomy is not considered necessary | Huibers et al | 4.11 | 5.22 |
|  | 5-Alpha reductase inhibitor with symptomatic prostatism, where prostatectomy is not considered necessary or can be postponed | Huibers et al | 2.03 | 2.57 |
|  | Topical vaginal oestrogen or vaginal oestrogen pessary for symptomatic atrophic vaginitis | Huibers et al | 0.00 | 0.00 |
| H. Analgesics | High-potency opioids (exception methadone) in moderate-severe pain, where paracetamol, NSAIDs or low-potency opioids are not appropriate to the pain severity or have been ineffective | Huibers et al | 0.01 |  |
|  | Short working opioids for break through pain with treatment of long working opioids | Not coded  *Data for “break through pain” not available* | - | - |
|  | Laxatives in patients receiving opioids | Huibers et al | 17.26 | - |
| I. | Seasonal trivalent influenza vaccine annually | Huibers et al | 0.00 | 0.00 |

SSRI = Selective Serotonin Re-uptake Inhibitors, SNRI = Selective Serotonin and Noradrenalin Reuptake Inhibitor , ACE= Angiotensin-Converting Enzyme, NSAID= Non-Steroidal Anti-Inflammatory Drugs, PPI = Proton Pump Inhibitor, DMARD = Disease-modifying anti-rheumatic drug, T-score = Bone Mineral Density T-score

* ATC codes, ICD-9 and ICD-10 codes required for Dutch STOPP/START v2 were selected using Huibers et al or selected - by two researchers (BD and KR) - using [www.whocc.no/atc_ddd_index/](http://www.whocc.no/atc_ddd_index/), the Dutch medication information website [www.farmacotherapeutischkompas.nl/](http://www.farmacotherapeutischkompas.nl/), [www.icd9data.com](http://www.icd9data.com), [www.icd10data.com](http://www.icd10data.com) or diagnoses description in our data.

** Dutch STOPP/START criteria v2 with diagnoses were calculated over the whole admission. Criteria with only medications were calculated based on the medications administration dates and we identified a START violation if two medications were not administrated on the same day (exception is methotrexate). We excluded medication administrations with missing values or status “planned” (indicating that the medication had not been given).

*** Prevalence (% time) was calculated as percentage of a selected population (i.e. female/male or admissions with a selected length of stay: ≥4 weeks in “Benzodiazepines for ≥ 4 weeks”).

**Appendix 2:** Time trends for all PIMs and PPOs and for the top 5 most prevalent PIMs and PPOs.

| Table A3. Time trends (per year) of all PIMs and PPOs and top 5 PIMs and PPOs | | | | |
| --- | --- | --- | --- | --- |
|  | Crude OR  (95%) | P-value | Adjusted OR (95%) | P-value |
| **PIMs (all)** | **1.00**  **(1.00 – 1.00)** | **0.95** | **0.91**  **(0.91-0.91** | **<0.001** |
| 1. Benzodiazepines with history or risk of falling | 0.82  (0.82 – 0.82) | <0.001 | 0.78  (0.78 – 0.78) | <0.001 |
| 1. Loop diuretic as treatment for hypertension | 1.14  (1.14 – 1.14) | <0.001 | 1.05  (1.05 – 1.05) | 0.01 |
| 1. Neuroleptic drugs with history or risk of falling | 0.83  (0.83 – 0.83) | <0.001 | 0.80  (0.80 – 0.80) | <0.001 |
| 1. Antiplatelet agents with vitamin K antagonist, direct thrombin inhibitor or factor Xa inhibitors in patients with stable coronary, cerebrovascular or peripheral arterial disease | 0.97  (0.97– 0.97) | 0.32 | 0.90  (0.90 – 0.90) | <0.001 |
| 1. Centrally-acting antihypertensives | 1.05  (1.05 – 1.05) | 0.10 | 1.12  (1.12 – 1.12) | <0.001 |
| **PPOs (all)** | **1.08**  **(1.08** – **1.08)** | **<0.001** | **0.94**  **(0.94** – **0.94)** | **<0.001** |
| 1. Laxatives in patients receiving opioids | 0.97  (0.97 – 0.97) | 0.11 | 0.95  (0.95 – 0.95) | 0.02 |
| 1. Proton Pump Inhibitor with a low dose acetyl salicylic acid or carbasalate calcium (and age specific criteria) | 0.99  (0.99 – 0.99) | 0.55 | 0.93  (0.93 – 0.93) | <0.01 |
| 1. ACE inhibitor (or angiotensin receptor blocker in case of side effects ACE inhibitor) with systolic heart failure and/or coronary artery disease | 1.06  (1.06 – 1.06) | <0.01 | 0.92  (0.92 – 0.92) | <0.001 |
| 1. Statin therapy with a documented history of coronary, cerebral or peripheral vascular disease or high cardiovascular risk and LDL > 2,5 mmol/l, unless the patient’s life expectancy < 3 years. | 1.07  (1.07 – 1.07) | <0.01 | 0.96  (0.96 – 0.96) | 0.09 |
| 1. Appropriate beta-blocker with stable systolic heart failure | 1.08  (1.08 – 1.08) | <0.01 | 0.87  (0.87 – 0.87) | <0.001 |
